# Impact of perioperative blood transfusion on clinical outcomes in patients with colorectal liver metastasis after hepatectomy: a meta-analysis

**DOI:** 10.18632/oncotarget.16771

**Published:** 2017-03-31

**Authors:** Xinghua Lyu, Wenhui Qiao, Debang Li, Yufang Leng

**Affiliations:** ^1^ Department of Anaesthesiology, The First Hospital of Lanzhou University, Lanzhou, China

**Keywords:** colorectal, liver, oncology, outcomes

## Abstract

**BACKGROUND:**

Perioperative blood transfusion may be associated with negative clinical outcomes in oncological surgery. A meta-analysis of published studies was conducted to evaluate the impact of blood transfusion on short- and long-term outcomes following liver resection of colorectal liver metastasis (CLM).

**MATERIALS AND METHODS:**

A systematic search was performed to identify relevant articles. Data were pooled for meta-analysis using Review Manager version 5.3.

**RESULTS:**

Twenty-five observational studies containing 10621 patients were subjected to the analysis. Compared with non-transfused patients, transfused patients experienced higher overall morbidity (odds ratio [OR], 1.98; 95% confidence intervals [CI] =1.49-2.33), more major complications (OR, 2.12; 95% CI =1.26-3.58), higher mortality (OR, 4.13; 95% CI =1.96-8.72), and longer length of hospital stay (weighted mean difference, 4.43; 95% CI =1.15-7.69). Transfusion was associated with reduced overall survival (risk ratio [RR], 1.24, 95% CI =1.11-1.38) and disease-free survival (RR, 1.38, 95% CI=1.23-1.56).

**CONCLUSION:**

Perioperative blood transfusion has a detrimental impact on the clinical outcomes of patients undergoing CLM resection.

## INTRODUCTION

Colorectal cancer is the third most common malignancy worldwide with approximately 50% patients developing liver metastasis during the course of disease. Hepatic resection represents potentially curative treatment for colorectal liver metastasis (CLM) and offers an opportunity of long-term survival benefit, with 5-yeart overall survival (OS) rate of 37-58% [1]. Although advances in surgical techniques and perioperative care have decreased the morbidity and mortality remarkably in high-volume centers, a considerable proportion of patients have to receive perioperative blood transfusion (PBT) [2–4]. Transfusion-related immune modulation may compromise the clinical outcomes in oncological surgery. However, data for evaluating the impact of PBT on short- and long-term outcomes following CLM resection are limited due to small sample sizes in most reported studies [2–10]. In this study, we made a meta-analysis on the presently existing data in the literature to assess this issue.

## RESULTS

### Selection of studies

**Figure 1:**
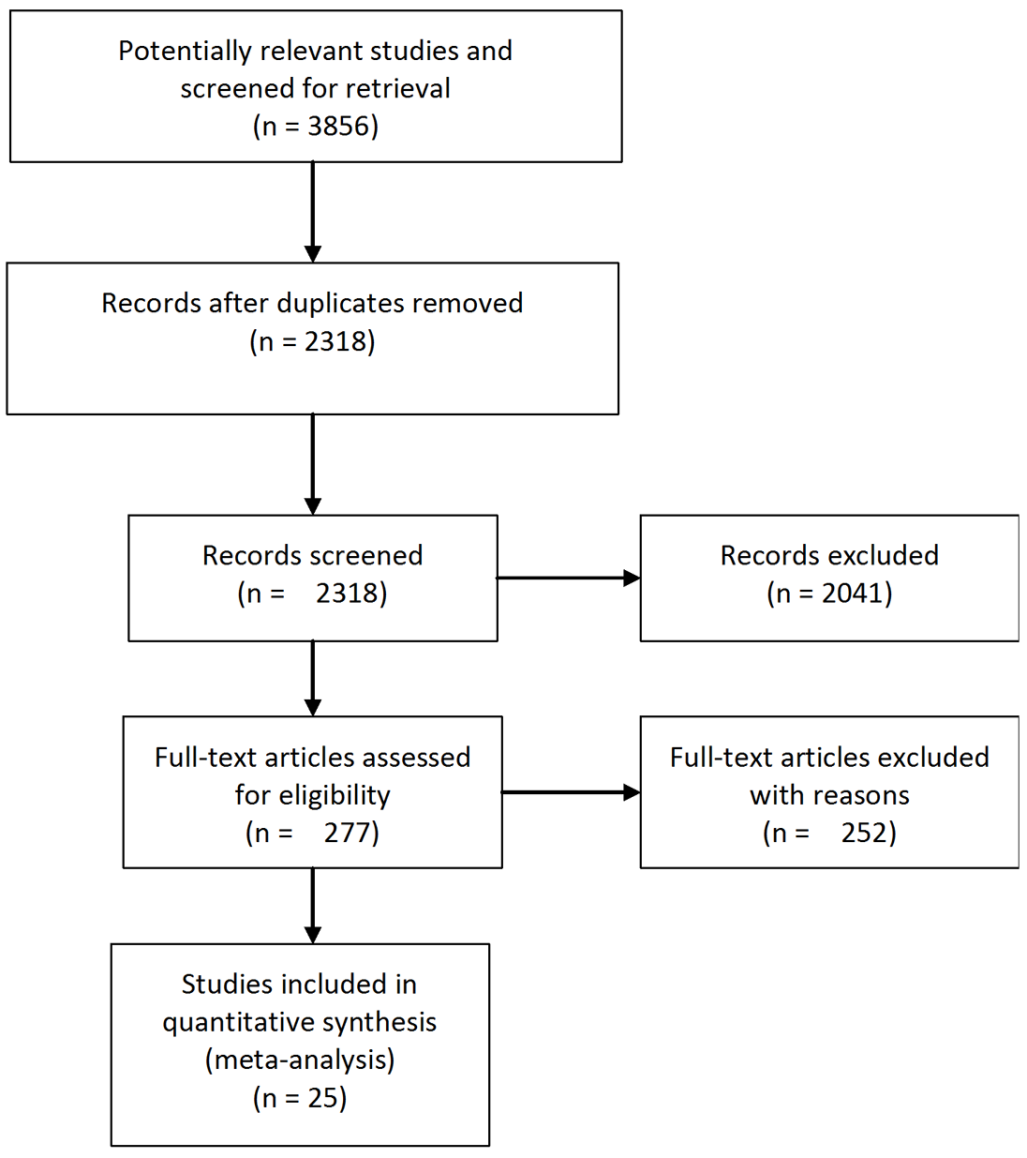
Flowchart of study selection

The initial search yielded 3856 articles, of which 25 published between 1988 and 2017 were finally qualified for the inclusion criteria in the meta-analysis [2–9, 11–26]. The process of study selection is shown in Figure 1. Study characteristics are shown in Table 1. Two articles from the same institution were included [5, 9], the former mainly assessing the impact of transfusion on perioperative morbidity and mortality, and the latter mainly assessing the impact of transfusion on long-term survival. All identified studies were observational design studies involving a total of 10621 patients. Seven studies were from USA [5, 7, 9, 16, 21, 22, 24], four from Italy [4, 8, 12, 13], three from Japan [3, 14, 18], two from UK [11, 25], two from Germany [20, 23], one from Sweden [2], one from France [6], one from Spain [10], one from Brazil [15], one from China [17], one from Canada [19], and one from the Netherlands [26]. The blood product transfusion rate was highly variable across studies ranging from 13.5% to 91.5%. The sample size of each study varied from 65 to 1351 patients.

**Table 1:** Clinical background of studies included in the meta-analysis

Reference	Year	No. of patients	PT (%)	M/F	Age, years	TS,cm	No. of Tumor	Morbidity (%)	Mortality (%)	5 y-OS (%)	5 y-DFS (%)	Study quality
Ohlsson [2]	1998	111	91.5	60/51	≥ 65, n=75	≥ 5, n=54	≥ 2, n=50	17.1	3.6	25	19	6
Ambiru [3]	1999	168	77.4	104/64	62 (21-80)	≥ 5, n=56	≥ 4, n = 38	29.7	3.5	26	NA	6
Ercolani [4]	2002	245	43.0	144/102	> 60, n=115	NA	≥ 3, n=41	18.7	0.8	34	NA	7
Kooby [5]	2003	1351	54	772/579	NA	NA	NA	40	3.7	36	NA	8
Laurent [6]	2003	311	15.7	209/102	63 (31–86)	≥5, n=142	> 3, n=42	29.9	2.8	36	24	9
Zakaria [7]	2007	662	55.2	404/258	60 ± 11	NA	NA	NA	2.8	42	NA	7
Arru [8]	2008	297	53.2	171/126	≥ 65, n=120	> 5, n=98	≥ 2, n=117	17.0	NA	27.5	NA	9
Ito [9]	2008	1067	44.6	596/471	61 ± 0.37	5.0 ± 3.7	3.4 ± 0.05	42.2	Excluded	41	25	7
Hernández [10]	2009	210	24.2	140/70	61 ± 12	≥ 5, n=80	≥ 3, n=67	42.9	1.4	53.8	23	7
Farid [13]	2010	705	21.1	442/263	46 (23–91)	4 (0.1–23)	3 (1–21)	7.9	3.5	34	22	8
Giuliante [14]	2010	543	23	309/234	62 (24–83)	4.5 ± 3.0	2.0 (1–14)	18.5	1.3	36.5	25.7	7
Gruttadauria [15]	2011	127	40.2	72/55	63 (55–69)	NA	NA	47.2	NA	NA	NA	8
Kaibori [16]	2012	119	37.8	70/49	>64, n=62	>3.5, n=59	≥3, n=38	22.6	0	38.7	33.7	8
Ribeiro [17]	2012	170	31.7	91/79	59 (23-80)	> 5, n=72	≥ 3, n=64		2.9	64.9	39.1	7
Cannon [18]	2013	239	26.8	NA	61.4	4.6	-	47.3	2.5	32.7	18.1	8
Jiang [19]	2013	139	25.8	91/48	58 (25–82)	2.5(0.3–11.5)	≥ 2, n=66	12	0	53	48	9
Shiba [20]	2013	65	41.5	45/20	64.1±10.0	NA	1.8 ±2.1	29.2	NA	46.7	NA	7
Hallet [21]	2015	483	27.5	299/184	NA	NA	NA	NA	4.8	56.8	27.0	9
Schiergens [22]	2015	292	36.3	193/99	65 (21-86)	> 5, n=52	≥ 3, n=43	40	5	49	49	9
Postlewait [23]	2016	456	30.7	252/204	58.6 ± 12.0	4.6 ± 3.2	1.9 ± 1.2	21.2	1.3	36.5	NA	7
Zimmitti [24]	2016	510	17.6	309/201	57 (23–87)	2.3 (0.3–11.5)	1 (0–80)	40.4	Excluded	56.6	31.6	7
Kulik [25]	2016	983	52.2	605/378	≥ 70, n=235	> 5, n=377	≥ 2, n=501	17.7	1.2	NA	NA	6
Margonis [26]	2016	433	13.5	255/178	54 (44–64)	2.8 (1.7–4.5)	5 (2–7)	NA	NA	49.3	NA	6
Bell [27]	2017	727	13.5	466/261	64 (25–88)	> 5, n=270	> 3, n=281	26	4.5	NA	NA	6
Olthof [28]	2017	208	NA	136/72	64 (56–71)	3.1 (2–5.1)	2 (1–3)	36	1	59	29	8

Abbreviations: PT= perioperative transfusion, M= male, F=female, TS=tumor size, NA=not available; OS=overall survival; DFS=disease-free survival

### Meta-analysis

Table 2 shows the results for the outcomes.

**Table 2:** Meta-analysis of short and long-term outcomes

Outcome of interest	Studies	Participants	OR/WMD	95% CI	*P*-value	*I*^2^ (%)
Clinicopathologic features						
Male gender	3	1231	0.58	0.46, 0.75	<0.001	0
Age	4	1470	0.10	-1.28, 1.47	0.10	40
Body mass index	2	695	-0.26	-1.26, 0.74	0.62	0
Preoperative anemiaa	2	775	2.51	1.83, 3.45	<0.001	0
ASA > 2	2	748	1.15	0.59, 2.24	0.69	76
Extended or major resection	4	1470	1.64	1.28, 2.09	<0.001	0
Duration of surgery (min)	2	775	55.64	42.14, 69.14	<0.001	0
Blood loss (mL)	4	1470	726.88	376,91, 1076.84	<0.001	93
Tumor size (cm)	2	695	0.95	-0.13, 2.03	0.09	78
Tumor number	2	695	-0.03	-0.49, 0.44	0.90	59
Negative surgical margin	4	1470	1.03	0.70, 1.51	0.89	0
Postoperative outcomes						
Overall morbidity	6	2833	1.98	1.49, 2.33	<0.001	51
Major complication	4	2226	2.12	1.26, 3.58	0.005	76
Mortality	5	2821	4.13	1.96, 8.72	<0.001	51
Length of stay (day)	3	2099	4.42	1.15, 7.69	0.008	82
Long-term outcomes						
Overall survival	21	8732	1.24	1.11, 1.38	0.0002	71
Disease-free survival	11	5018	1.38	1.23, 1.56	<0.001	17

Abbreviations: OR=odds ratio, WMD=weighted mean difference, CI= confidence interval,

ASA= American Society of Anesthesiologists

Four studies compared the characteristics of transfused patients versus those nontransfused patients [16, 19–21]. Pooled analysis showed that transfusion was associated with female gender (*P* <0.001), higher prevalent preoperative anemia (*P* <0.001), more extended or major hepatectomy (*P* <0.001), increased estimated blood loss (*P* <0.001), and longer duration of surgery (*P* <0.001).

**Figure 2:**
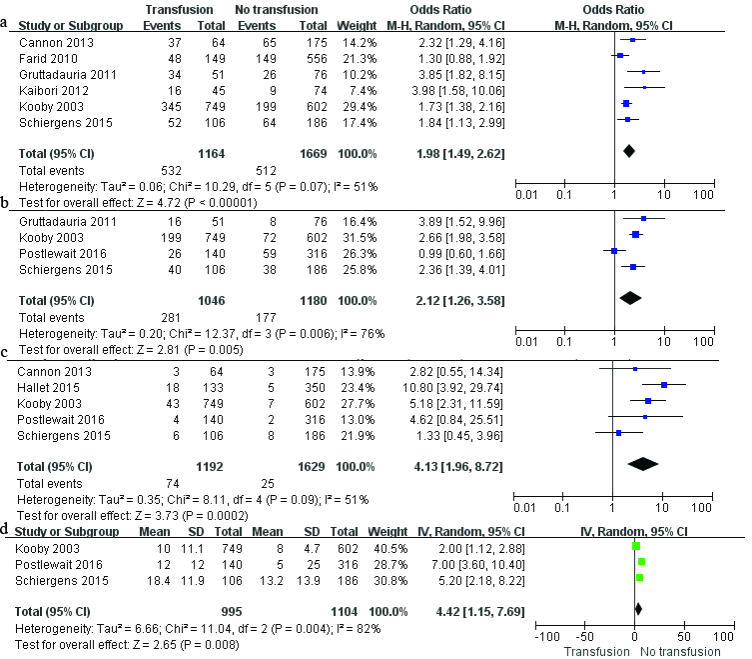
Results of the meta-analysis on perioperative outcomes **a**. overall morbidity; **b**. major complications; **c**. mortality; and **d**. lengths of hospital stay.

The impact of PBT on perioperative outcomes was evaluated in 6 studies [5, 11, 13, 14, 16, 20]. As shown in Figure 2, compared with nontransfused patients, transfused patients experienced higher overall morbidity (*P* <0.001), more major complication (Clavien-Dindo class 3–5 [27]) (*P* =0.005), higher mortality (*P* <0.001), and longer lengths of hospital stay (*P* <0.001).

**Figure 3:**
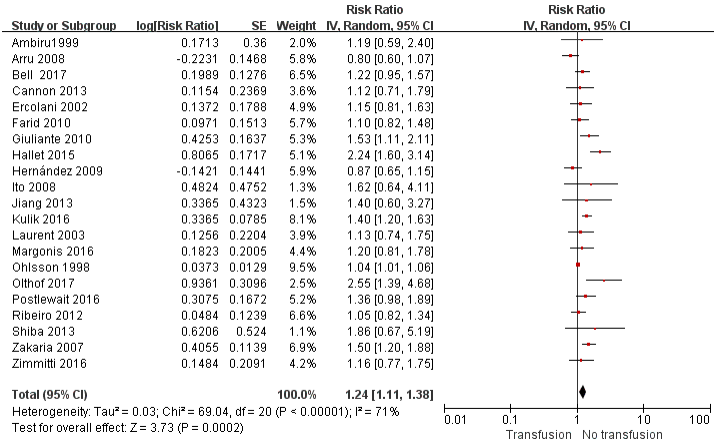
Results of the meta-analysis on overall survival

**Figure 4:**
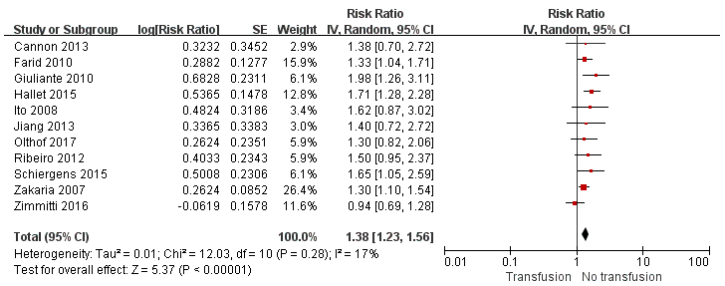
Results of the meta-analysis on disease-free survival

The impact of PBT on OS and disease-free survival (DFS) was evaluated in 21 [2–9, 11, 12, 15–19, 21–26] and 11 [6, 8, 11, 12, 15–17, 19, 20, 22] studies, respectively. The 5-year OS and DFS of transfused patients ranged from 21.5% to 62.7% and 14.7% to 42% respectively *vs*. 24–66.2% and 19.5–55% in nontransfused patients. Pooled analysis showed that transfusion correlated with poor OS (*P* =0.0002) (Figure 3) and DFS (*P* <0.001) (Figure 4). The summary of risk ratio (RR) estimates by multivariate analysis was 1.37 (95% confidence intervals [CI] =1.12–1.68; *P* =0.002) in 11 studies [2, 6–8, 12, 17–19, 24–26] for OS, and 1.40 (95% CI =1.25–1.58; *P* <0.001) for DFS in six studies [6, 8, 11, 17, 19, 20]. In sensitivity analysis, removing of any single study from the analysis did not affect the overall results regarding the negative association between transfusion and long-term survival (data not shown).

There was significant heterogeneity between studies (I^2^ =71%) regarding the impact of PBT on OS. In meta-regression analysis, year of publication, sample size, and country of patients were significant sources of heterogeneity (Table 3).

**Table 3:** Meta-regression analysis between pooled relative risk and co-variates of overall survival

Co-variates	Coefficient	95% CI	Std. Err.	*P*-value
Year of publication	0.2546	0.1310, 0.3783	0.0631	<0.001
Sample size	0.1655	0.0296, 0.3015	0.0694	0.017
Country of patients	0.1160	0.0953, 0.1367	0.0106	<0.001

Abbreviations: CI =confidence interval

### Publication bias

**Figure 5:**
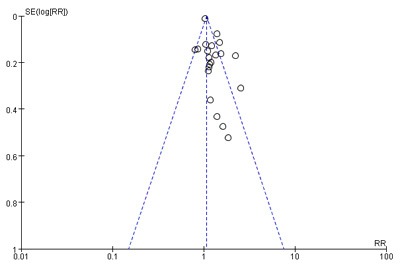
Funnel plot for the results from overall survival

A funnel plot reveals asymmetry for the effect of PBT on OS indicating the presence of publication bias (Figure 5).

## DISCUSSION

While blood transfusion is important in maintaining hemodynamic stability and end organ perfusion during complex surgeries, it still carries significant risks, including incompatibility, transmission of infectious agents, coagulopathy, allergic reactions, and tumor-promoting action [30]. Since Burrows and Tartter first reported that PBT may worsen the postoperative survival of patients with bowel cancer in 1982 [31], a large number of authors have investigated the impact of PBT on clinical outcomes in patients with cancer subjected to surgery. In the field of hepatopancreaticobiliary oncological surgery, a meta-analysis of 23 studies reported that patients receiving PBT had significantly lower 5-year survival after curative-intent pancreatic surgery (OR, 2.43, 95% CI =1.90–3.10) [32]. Another meta-analysis of 22 studies noted that hepatocellular carcinoma patients receiving PBT had an increased risk of all-cause death at 3 and 5 years after surgery (respectively: OR = 1.92, 95% CI, 1.61-2.29; OR = 1.60, 95% CI, 1.47-1.73) compared with those without PBT [33]. In contrast, the evidence is less clear in CLM surgery. To the best of our knowledge, this is the first meta-analysis that selectively focused on surgical CLM populations. The result clearly indicates that PBT compromised long-term survival dramatically.

Beyond its deleterious effect on long-term outcomes after surgery, PBT is also associated with adverse perioperative sequelae as measured by overall morbidity, major complications, mortality, and length of stay in the current study. More specifically, more occurrences of postoperative infection or liver failure were observed in patients receiving PBT [6, 11]. In a recent review of 712 consecutive elective hepatectomy (all diseases), Hallet et al. [34] found that PBT was associated with an increased rate of major complications and a longer length of hospital stay. The observations from non-hepatic surgery also demonstrated similar results [35, 36].

An important issue is whether the association between PBT and the outcome variables analyzed represents a causative effect or whether there are unmanageable confounders acting inwardly. It can be presumed certain that the transfused patients may represent a compromised and vulnerable cohort, and poor outcomes may be attributed to other factors associated with PBT unless otherwise further confirmed by a multivariate model [20]. Indeed, the results of our pooled data of multivariate RR are similar to the findings from overall analysis regarding long-term survival. Although we were unable to pool multivariate RR for perioperative outcomes due to insufficient data, in one included study, PBT was identified at multivariate analysis as a significant predictor of overall morbidity, major complications and mortality after other variables were adjusted [5]. Therefore, there are risks linked to poor postoperative outcomes inherently associated with transfusion *per se* rather than a confounder.

The mechanism underlying the detrimental effect of PBT on postoperative outcomes after oncologic surgery remains to be elucidated. One possible reason is the immunosuppressive effect of transfusion. The observed alterations include suppression of cytotoxic cells and monocyte activity, release of immunosuppressive prostaglandins, inhibition of interleukin-2 production, and increase in suppressor T-cell activity [30].

This meta-analysis has several potential limitations. First, all included studies are observational studies that provided a low level of evidence. Studies may have differed with regard to the baseline characteristics of the patients, tumor size or disease stage, operative procedures, the amount of blood loss, adjuvant treatment, and the follow-up duration. The results therefore are susceptible to heterogeneity. Second, the timing or amount of transfusion received was not taken into account because most of these published studies lacked relevant information. Finally, the review was restricted to articles published in English. This selection could favor the positive studies, as positive results tend to be published in English-language journals, while negative studies tend to be reported in native languages. There is therefore a publication bias.

In conclusion, PBT has a detrimental impact on clinical outcomes in patients undergoing CLM resection. Both surgeons and anesthesiologists need to manage perioperative care from various aspects to minimize the use of transfusion.

## MATERIALS AND METHODS

This study was done in accordance with the recommendations of the preferred reporting items for systematic reviews and meta-analyses (PRISMA) [28].

### Study selection and criteria for inclusion

A systematic search of PubMed, Science Citation Index, and Embase databases was performed to identify relevant articles from the time of inception to March 2017 using the following key words: colorectal liver metastases, liver resection, and transfusion. Manual search of reference lists of all retrieved articles was carried out to identify additional studies.

Original publications in the English language examining the impact of PBT on the on short- and long-term outcomes following CLM resection were eligible. Letters, reviews, abstracts, editorials, expert opinions, non-English language papers, duplicated studies, and animal studies were excluded.

### Data extraction and outcomes of interest

Two reviewers (XL and YL, respectively) independently extracted relevant data regarding the characteristics of study and outcomes of interest from each selected article by using standardized data extraction forms. Discrepancies were resolved through discussion and consensus.

The outcomes of interest were clinicopathologic characteristics, postoperative morbidity and mortality, OS and DFS.

### Assessment of methodological quality

The methodological quality of included studies was assessed by using the Newcastle-Ottawa Scale. Scores are assigned for patient selection, comparability of the study groups, and outcome assessment [29].

### Statistical analysis

The effect measures estimated were odds ratios (OR) 95% CI for dichotomous variables and weighted mean difference (WMD) with a 95% CI for continuous data. The RR with 95% CI was used to assess the prognostic value of transfusion. The I^2^ statistic was calculated to assess the heterogeneity in results across studies with values>50% representing substantial heterogeneity. A funnel plot based on the OS was used to detect the possibility of publication bias. Sensitivity analyses were carried out to investigate the impact of individual study on the overall outcome of the meta-analysis. Meta-regression was performed with the following co-variates: sample size, year of publication, and country of patients. Statistical analyses were performed with Review Manager version 5.3 (The Cochrane Collaboration, Software Update, Oxford) and Stata™ version 8.0 (Stata Corporation, College Station, Texas, USA). Values of *P* < 0.05 were considered statistically significant.

## SUPPLEMENTARY MATERIALS



## Abbreviations

CLM: colorectal liver metastasis
OS: overall survival
PBT: perioperative blood transfusion
OR: odds ratios
CI: confidence intervals
WMD: weighted mean difference
RR: risk ratio.
